# GRASP: Guided Reference-based Assembly of Short Peptides

**DOI:** 10.1093/nar/gku1210

**Published:** 2014-11-20

**Authors:** Cuncong Zhong, Youngik Yang, Shibu Yooseph

**Affiliations:** Informatics Department, J. Craig Venter Institute, La Jolla, CA 92037, USA

## Abstract

Protein sequences predicted from metagenomic datasets are annotated by identifying their homologs via sequence comparisons with reference or curated proteins. However, a majority of metagenomic protein sequences are partial-length, arising as a result of identifying genes on sequencing reads or on assembled nucleotide contigs, which themselves are often very fragmented. The fragmented nature of metagenomic protein predictions adversely impacts homology detection and, therefore, the quality of the overall annotation of the dataset. Here we present a novel algorithm called GRASP that accurately identifies the homologs of a given reference protein sequence from a database consisting of partial-length metagenomic proteins. Our homology detection strategy is guided by the reference sequence, and involves the simultaneous search and assembly of overlapping database sequences. GRASP was compared to three commonly used protein sequence search programs (BLASTP, PSI-BLAST and FASTM). Our evaluations using several simulated and real datasets show that GRASP has a significantly higher sensitivity than these programs while maintaining a very high specificity. GRASP can be a very useful program for detecting and quantifying taxonomic and protein family abundances in metagenomic datasets. GRASP is implemented in GNU C++, and is freely available at http://sourceforge.net/projects/grasp-release.

## INTRODUCTION

Metagenomics is a cultivation-independent paradigm for studying microbes and involves the shotgun sequencing of DNA extracted from biological samples that are collected from the environment of interest (1). It has greatly furthered our understanding of microbial diversity and ecology, and has broad applications in many fields including human health (2–5), ecological and environmental surveys and monitoring (6–8), and renewable energy (9–11). Next-generation sequencing (NGS) technologies (12–15) are used routinely to sequence metagenomic samples and provide a cost-effective approach to study the genomic content of the constituent microbes at high sequence coverage.

In addition to allowing for the inference of taxonomic composition of the community, metagenomic sequence data can also be used to reconstruct the functional and metabolic potential of the organisms that are present. This requires the identification of proteins that are present in the metagenomic sample. Accurate *de novo* gene finders are available (16,17) that enable the identification of protein sequences directly from NGS reads or from assemblies of these reads. However, the short length of reads from current NGS technologies implies that most of the protein predictions will be fragmentary (referred to as short peptides). The quality of nucleotide assemblies is influenced by the diversity of the community, and assemblies are often very fragmented (3,18–21); subsequently, protein predictions on assembled contigs or scaffolds also result in a large number of partial-length sequences. The fragmented nature of protein predictions from metagenomic datasets has consequences for the downstream annotation of proteins (i.e. for the assignment of protein name and function).

The process of annotating a metagenomic protein sequence proceeds via identification of its homologs in comparison with reference protein sequences (22–25), using a sequence comparison program (26,27). However, the sensitivity and specificity of this approach can be adversely impacted by the short length of the metagenomic protein sequence and is also dependent on the degree of conservation of the region in the protein family that the sequence belongs to. Here we present a novel approach that can significantly improve the homolog identification process and, therefore, also improve annotation. Our approach is based on the observation that shotgun sequencing generates reads that are distributed randomly across the constituent genomes. With increased sequencing depth, many of these reads will belong to overlapping genomic regions; this implies that there will be overlapping short peptide sequences in the metagenomic dataset. Therefore, an ‘assembly’ of these overlapping sequences during sequence comparison should improve homology detection; furthermore, an assembly of the short peptide sequences should be more effective (compared to nucleotide read assembly) since amino acid conservation extends over a great taxonomic range compared to nucleotide conservation (28). The computational problem is thus formulated as one where we are given a reference protein sequence (e.g. from a curated protein database) as query and a database of short peptide sequences (from a metagenomic dataset), and our goal is to identify the homologs of the reference sequence in this database. Our solution to this problem is a reference sequence-guided approach that involves the simultaneous assembly and search of sequences in the database; our algorithm is called GRASP (Guided Reference-based Assembly of Short Peptides).

**Figure 1.**
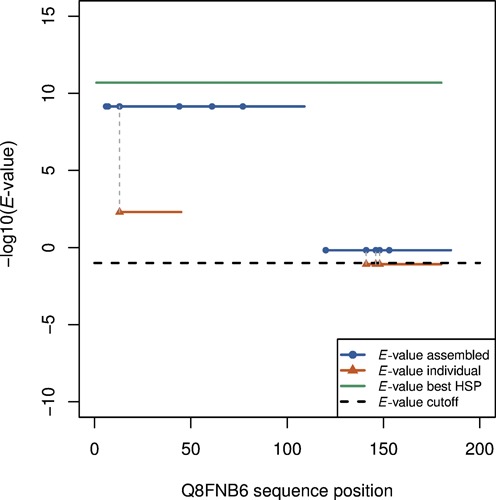
An example showing the improved sensitivity of homology detection when the individual reads are assembled prior to alignment. BLASTP alignment between the query (1VDX_A) and the full-length sequence for Q8FNB6 identifies high-scoring pair (HSP) spanning a majority of this sequence (green segment). For reads (red segments) sampled from Q8FNB6, only one out of 11 can be identified (above the default BLASTP cutoff, dashed black line). However, if the reads are assembled based on overlap, all of them can be identified as homologs as they form part of contigs (blue segments) with improved *E*-values.

Figure 1 shows an example highlighting the improved sensitivity of detecting homologs when overlapping database sequences are ‘assembled’, in contrast to the strategy where the reference sequence is searched ‘independently’ against each database sequence. For this example, a database of short peptides was constructed using a member (Q8FNB6) of the LigT like Phosphoesterase protein family (Pfam PF02834 (29)), by randomly sampling short peptide fragments from this sequence at 2X coverage. Another member (1VDX_A) of the same family was used as the query sequence, and NCBI's BLASTP program (27) was used to search for homologous sequences in this database. It is observed that the majority of the individual short peptides (red segments) were not identified—of the 11 database sequences, only one had a match above threshold, while for seven sequences, no seed was detected to even initiate alignment. This result indicates the low sensitivity of detecting homologs when short fragments are present and searched independently, even though the full-length sequences for the two members align with a very good *E*-value (green segment). By assembling the overlapping individual short peptides into longer contiguous sequences (blue segments), the *E*-value of the resulting alignments can be significantly improved; in this example, all 11 reads contribute to one of the two assembled sequences (blue segments).

GRASP was implemented using GNU C++ and its performance was compared to that of three commonly used sequence homology detection programs (NCBI's BLASTP (27) and PSI-BLAST (30) programs, and the FASTM program (31)) that search a given reference (or query) protein sequence against a database of amino acid sequences. Both simulated and real datasets were used in the evaluations. The results show that, for the same specificity, GRASP is capable of improving the sensitivity by ∼20% on the Pfam-based simulated datasets. For the simulated metagenomic dataset, the benchmark results show that GRASP can improve the sensitivity by ∼10% over the other programs at the same specificity level. For the real dataset, GRASP can identify approximately twice more homologs than the other search programs at an *E*-value cutoff of 10 (with an expected specificity >99%), while at a more stringent *E*-value cutoff of 10^−10^, GRASP is able to identify three times more homologs than FASTM, and at least five times more homologs than BLASTP and PSI-BLAST. The GRASP implementation is freely available at http://sourceforge.net/projects/grasp-release.

## MATERIALS AND METHODS

Let *Q* denote the reference (or query) protein sequence and let *R* denote the database consisting of short peptide sequences; these short peptides will henceforth be referred to as reads. Our goal is to identify a set of contigs where each contig sequence has an alignment with *Q* that meets a user-specified alignment threshold (*E*-value). Each contig is either a single read, or it is an assembly of overlapping reads, in which case, each constituent read is a substring of the contig sequence. In our framework, a read may be assigned to multiple contigs. However, to avoid redundancy in the output, the set of contigs that is produced by GRASP is such that no contig is a substring of another contig in the set.

Conceptually, the GRASP algorithm can be thought of as a series of traversals of the overlap graph constructed from the reads, where each traversal is carried out using the query sequence as a guide so as to identify paths in the graph that have high scores when aligned with the query sequence. In theory, this overlap graph can be defined as in traditional overlap-consensus-layout assemblers (32), where each node in the graph corresponds to a read and there is a directed edge between two nodes (reads) if the suffix of one read is the prefix of the other. Alternatively, the overlap graph can be a more compact representation of read overlaps defined using *k*-mers that are shared between reads (33); this definition is often more tractable for large NGS datasets. Important components of the graph traversal strategy include the identification of regions in the graph where path traversal can be initiated, rules for path extension and criteria for traversal termination. In the GRASP algorithm, however, the overlap graph is not explicitly constructed. Instead, a suffix array (34) data structure is used to capture read overlap information and is used to identify reads that can be used to extend the current path. The suffix array is a space efficient data structure that represents all suffixes of a given text in a lexicographically sorted fashion, thus enabling an efficient determination of the occurrence(s) of a query pattern in this text.

**Figure 2.**
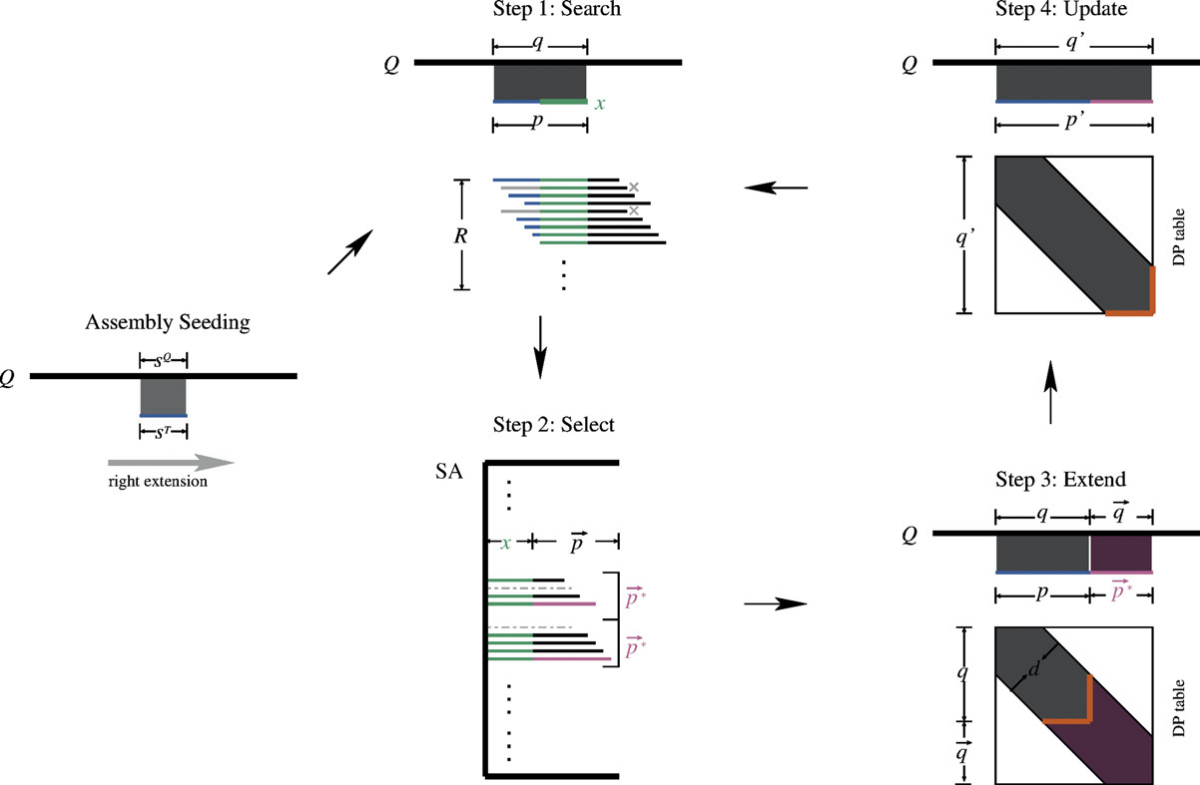
The GRASP algorithmic framework for right extension. First, a seed pair $(s^Q ,s^T )$ is identified by perfect $k$-mer matches in the reduced amino acid alphabet space (Assembly Seeding). For each pair, subsequent extension steps are performed iteratively (implemented using a priority queue). In Step 1, all supporting reads for extending path $p$ are identified through suffix array searches. Supporting reads for $p$ are those containing both green and blue segments; these reads are identified by querying $r{\rm SA}$ using $rev(x)$ (see main text for details). In Step 2, the maximal extension sequences $\vec p^*$ (marked red) are selected from the set of supporting reads. In Step 3, the maximal extension sequences are used to extend the assembly from $p$ to $p\prime$. Banded alignment of $p\prime$ and the extended query substring $q\prime$ is computed by referring to previously computed alignment scores at the boundary (orange). Only the red region in the dynamic programming (DP) table is filled. In Step 4, the extended alignment information is recorded and the priority queue is updated with new extension sequences.

The algorithmic framework of GRASP is outlined in Figure 2. Initially, a set of *k*-mer pairs is constructed from the query and database sequences, where the query sequence *k*-mer and the database sequence *k*-mer in a pair are highly similar to each other; each such *k*-mer pair initiates contig assembly (or equivalently, path traversal in the overlap graph). First, initiated by a *k*-mer pair, assembly proceeds in the C-terminus direction of the query sequence; this phase is referred to as ‘right extension’. In this phase, a set of (possibly overlapping) assembly paths is identified and maintained, along with a priority queue of candidate path extension sequences that are updated iteratively. The extension sequences are identified using reads that overlap with paths in the path set. Banded alignment is used to determine if an extended path has sufficient score when aligned with the query. The candidate at the head of the priority queue is used to identify the next path for extension, and subsequently, new candidate extensions that overlap with this extended path are noted and the priority queue is updated. Extension stops when a termination criterion is met. After right extension completes, initiated by the same *k*-mer pair, assembly proceeds in the N-terminus direction of the query sequence (referred to as ‘left extension’) in a similar manner as the right extension. At the end of this phase, paths from the right and left extensions that can be concatenated based on reads that bridge them and have resulting alignment *E*-values meeting the user-specified threshold, are identified. These concatenated paths constitute the GRASP output. The assembly process is parameterized, and includes parameters for identifying overlapping reads and for determining score drop-off during alignment. These parameters can be used to control the specificity and sensitivity of homolog detection.

### Notation

To facilitate the exposition of the GRASP algorithm, we define the basic notations that will be used. Let *m* be an arbitrary string derived from an alphabet Σ, and let |*m*| denote its length. Also, let *m_i_* denote the *i*^t^^h^ character of *m*, and *m_i,j_* denote the substring of *m* that begins with *m_i_* and ends with *m_j_*, inclusively. For $1 \le i \le j \le |m|$, we say that *m_i,j_* is a *prefix* of *m* if *i* = 1, and that *m_i,j_* is a *suffix* of *m* if $j = |m|$; an empty string can be the substring, prefix or suffix of any string. We use ‘·’ to denote the concatenation operation on strings. Define the reverse string of *m* as ${\rm rev}(m) = m_{|m|} \cdot m_{|m - 1|} \cdot ... \cdot m_2 \cdot m_1$, and for the read set *R*, define ${\rm rev}(R) = \{ {\rm rev}(r)|r \in R\}$.

We use SA to denote the generalized suffix array (34) that is built from *R* by concatenating all the reads (using a delimiter symbol that is not part of the alphabet). We use SA[*i*] to denote the *i*^th^ element of the suffix array, and SA[*i, j*] to denote the array range that begins with SA[*i*] and ends with SA[*j*], inclusively. A suffix array query returns an array range, where each suffix within the range shares the query sequence as a common prefix. Say SA[*i, j*] is returned by the query of a non-empty string *m*; then, for any *k* such that $i \le k \le j$, we have *m* as a prefix of SA[*k*], and SA[*k*] as a suffix of some $r \in R$. To facilitate efficient querying, we construct and store the Longest Common Prefix (LCP) information for adjacent suffixes in SA (i.e. ${\rm LCP}({\rm SA}[i - 1],{\rm SA}[i])$ for all $1 {<} i \le |{\rm SA}|$). We use *r*SA to denote a generalized suffix array constructed on rev(*R*).

For the right extension phase of the assembly algorithm, we consider a read *r* as a ‘supporting read’ for the extension of path *p* if some prefix of *r* is a suffix of *p* and the prefix length is ≥ *l* (a predefined length cutoff). For path *p* and a supporting read *r*, we use $p\prime = p \cdot \vec p_r$ to denote the extended path using *r*, where $\vec p_r$ is the ‘extension sequence’ (note that $\vec p_r$ is a non-empty suffix of the read *r*) for the current path *p*. Without confusion, we use $\vec p$ to represent an arbitrary extension sequence when its corresponding supporting read is not referred to explicitly. We denote the set of extension sequences for *p* as $\overrightarrow {PE} (p)$, i.e. $\overrightarrow {PE} (p) = \{ \vec p\}$. Correspondingly, for the query sequence, we have $q\prime = q \cdot \vec q$, where *q*, $\vec q$ and $q\prime$ are all substrings of the query sequence *Q* (note that *q* and $\vec q$ are adjacent substrings in *Q*).

### GRASP algorithm

#### Assembly seeding

Contig construction is initiated using *k*-mer pairs, where one *k*-mer of a pair belongs to the query sequence and the other *k*-mer to a database read sequence, and such that these *k*-mers are highly similar (as defined below). We use the concept of reduced amino acid alphabet (26,35,36) for the efficient identification of these *k*-mer pairs. It has been observed that many amino acids share similar physical and chemical properties, such that they contribute in a similar manner to protein folding and function. This observation has been used to partition the standard amino acid alphabet set Σ (where$|\sum | = 20$) into equivalence classes where all amino acids in an equivalence class share the same property and thus can be substituted by one designated member of the class. This substitution results in a reduced alphabet $\sum ^*$ of size less than 20; let $f:\Sigma \to \Sigma ^*$ be a mapping that denotes this substitution. Given the mapping $f:\Sigma \to \Sigma ^*$ and a string $m = m_1 \cdot m_2 \cdot ... \cdot m_{|m|}$, where each $m_i \in \sum$, we define ${\rm reduced}(m)$ to be the string $f(m_1 ) \cdot f(m_2 ) \cdot ... \cdot f(m_{|m|} )$.

We define MS to be the set of *k*-mer pairs $(s^Q ,s^T )$, where *s^Q^* is a *k*-mer of *Q, s^T^* is a *k*-mer of read *r* (for some $r \in R$). Each pair $(s^Q ,s^T )$ in MS is such that ${\rm reduced}(s^Q ) = {\rm reduced}(s^T )$ and the ungapped alignment score between *s^Q^* and *s^T^* is greater than a predefined threshold; alignment score is computed using an amino acid substitution matrix (e.g. BLOSUM or PAM). The set MS can be computed efficiently using a fast lookup function implemented using a hash-table containing all *k*-mers in $\sum ^*$ and their specific positions in the read set *R*. We used the reduced alphabet GBMR10 (26) for our evaluation of GRASP.

#### Right extension

Each pair in MS is used to initiate the construction of a set of assembly paths (or contigs), first by the process of right extension and then by the process of left extension. We describe the right extension phase here. In this phase, for a pair $(s^Q ,s^T )$, a set AS of assembly paths initiated by this pair and a priority queue PQ corresponding to the pair, are maintained; both sets are updated iteratively. The extensions initiated by $(s^Q ,s^T )$ are terminated when its priority queue becomes empty. Each path in AS is a contig under construction. Each element in PQ is a maximal extension sequence, derived from some supporting reads that can be used to extend some path from AS. The weight of each maximal extension sequence is the number of supporting reads that contain the sequence. For each path in AS, the corresponding coordinates on the query *Q* that it aligns to, are also stored along with the identifiers of the constituent reads.

The following steps are carried out in each iteration of the right extension phase. First, the head of PQ is used to identify a path in AS that can be extended and the new extended path is added to AS; let *p* denote this extended path and *q* denote the corresponding substring of *Q* that *p* aligns with. Next, the supporting reads for path *p* are identified by querying the suffix array (Step 1 in Figure 2). The supporting read set is pruned to remove those reads containing non-maximal extension sequences (Step 2 in Figure 2). For each of the remaining supporting reads, its corresponding suffix (i.e. extension sequence) is concatenated with *p*, in order to evaluate path extension. Each such extended path $p\prime$ is aligned with the extended query substring $q\prime$ (length determined by both $p\prime$ and the pre-set band size for the sequence alignment) to evaluate their sequence similarity (Step 3 in Figure 2). If the alignment score meets the threshold (which indicates that the assembly extension is desirable), this extension sequence is inserted into the priority queue using the weight of the extension sequence (Step 4 in Figure 2). If there are no extension sequences added to PQ for a path *p*, then *p* is not extended further; this happens when a termination criterion is encountered (described below). AS and PQ are initialized as follows: AS contains the sequence *s^T^*; PQ consists of supporting reads that are maximal extensions of this *k*-mer, and such that these extended sequences have alignment scores that meet the score threshold. Details of each of the four steps are presented below. The pseudo-code for the GRASP algorithm can be found in Supplementary Data B.

**Step 1: Identification of supporting reads using suffix array**: Supporting reads for the current path *p* can be obtained by querying the suffix array SA using all suffixes of *p* that are of length $ \ge l$, and, from each query result, then identifying reads whose prefixes are in the returned array range. However, using this approach, the entire SA has to be searched for each suffix of $p$ and can, in practice, be quite slow. Instead, in the GRASP algorithm, we use the reverse suffix array $r{\rm SA}$ to speed up the identification of supporting reads. Assume that $x$ is a string of length $l$ and is a suffix of $p$ (see Figure 2). First, the $r{\rm SA}$ is queried using ${\rm rev}(x)$; let $r{\rm SA}[i,j]$ denote the returned array range. From the reverse reads that are present in this array range, our goal then is to identify each reverse read whose suffix is a prefix of ${\rm rev}(p)$ (Figure 2, Step 1, reads with blue prefixes). This is accomplished by successively determining the next longest length prefix of ${\rm rev}(p)$ that is also a suffix of (one or more) reverse reads. Given the lexicographic ordering in a suffix array, we note that each of these determinations can be carried out by querying $r{\rm SA}$ in the array range $r{\rm SA}[i,j]$ using binary search (Supplementary Data B); this array range is much smaller than the full size of $r{\rm SA}$.
**Step 2: Identification of maximal extension sequences:** With the identification of the supporting reads for the current path $p$, the set $\overrightarrow {PE} (p)$ of possible path extensions for $p$, is also defined. However, for computational efficiency, it is unnecessary to consider every $\vec p \in \overrightarrow {PE} (p)$ to extend path $p$. Instead, we consider $\vec p^*$ as a candidate for extending $p$ only if it is a ‘maximal extension sequence’ (i.e. $\vec p^*$ is not a proper substring of any other $\vec p \in \overrightarrow {PE} (p)$, see Figure 2, Step 2, reads marked red). The supporting reads that give rise to maximal extensions can be identified as follows. First, the suffix array ${\rm SA}$ is queried using $x$, the length $l$ suffix of $p$, and the returned array range ${\rm SA}[i,j]$ is noted. This range also contains, and defines, a lexicographic ordering of all those suffixes (of supporting reads) that have $x$ as a prefix. Using the LCP array for ${\rm SA}$, it is also trivial to identify the LCP values of adjacent suffixes from the supporting reads in this ordering (34). If a suffix $\vec p_r$ [note that $\vec p_r \in \overrightarrow {PE} (p)$] of a supporting read $r$ is such that its LCP with the next suffix (of a supporting read) in this lexicographic ordering, is less than $|\vec p_r |$, then $r$ is designated as a supporting read generating a maximal extension and $\vec p_r$ is noted as a maximal extension sequence. This identification can be done in a single pass of the suffixes in the array range ${\rm SA}[i,j]$.
**Step 3: Banded alignment of extended path**: Suppose that the current path $p$, comprising of assembled reads, has been aligned to a substring $q$ of $Q$, and let $\vec p^*$ be a maximal extension sequence for $p$. It is desirable that the alignment is extended with some $\vec q$ in $Q$ that has a comparable length with $\vec p^*$; otherwise, the alignment could contain a large number of gaps, leading to a lower alignment score. Thus, the length of $\vec q$ is defined as $|\vec q| = |\vec p^* | + d/2$, where $d$ is the predefined band size (Figure 2, Step 3). For substring $q$, the extension $\vec q$ is uniquely defined given its length. For aligning sequences $p\prime = p \cdot \vec p^*$and $q\prime = q \cdot \vec q$, we do not compute the alignment of $p\prime$ to $q\prime$ from scratch. Instead, banded alignment scores are recorded at the end of each extension step (the last row and column, highlighted in orange in Figure 2), and subsequently used for the next extension. Thus, the banded alignment (Figure 2, Step 3, red region in the DP table) of the extension $\vec p^*$ and $\vec q$ are computed starting from the boundary scores for the banded alignment of $p$ and $q$. For this, we note that only the alignment scores in $O(d)$ cells in the dynamic programming table need to be recorded. This global alignment between $\vec p^*$ and $\vec q$, initiated using the boundary scores, is performed using a banded version of the Needleman–Wunsch algorithm (37).
**Step 4: Updates associated with alignment and path extension:** Required information for path extension includes the assembled path, the corresponding query substring and the boundary scores for the alignment between them (Figure 2, Step 4). Specifically, $p\prime$ is taken as the extended path, and correspondingly $q\prime$ is taken as the query substring. Then, the boundary scores for the alignment of $p\prime$ and $q\prime$ are stored (Figure 2, Step 4, highlighted in orange). The maximal extension sequences for $p\prime$ are inserted into PQ. The corresponding supporting reads are also recorded to facilitate trace back and also to detect termination of path extension.

#### Left extension

The left extension phase is analogous to the right extension phase. In the left extension, the read ${\rm rev}(r)$ is considered as a supporting read if some prefix of it is a suffix of ${\rm rev}(p)$, and the length of the prefix is at least *l*. Similarly, suppose *y* is the $l$-length prefix of $p$. The supporting reads are identified by querying ${\rm SA}$ using $y$, and the extension sequences are defined by querying $r{\rm SA}$ using ${\rm rev}(y)$. The assembly extension and alignment steps are performed in a similar fashion as for the right extension phase.

#### Termination criteria

The extension of a path $p$ stops if one of the following three conditions is met: sequence exhaustion, score drop-off and redundant extension. By sequence exhaustion, we mean that either the path alignment has reached one end of $Q$, or there is no supporting read for $p$. The score drop-off criterion is similar to that of BLAST—if the alignment score for the extended path is lower than the best score achieved so far (by a predefined threshold), then that extension is not considered. Finally, a redundant extension is defined as an extension whose supporting reads have been previously identified in some other path extensions and with the same alignment coordinates on the query $Q$. A hash table is maintained to record the assembled reads and the query positions where they are aligned. Supporting reads found in this hash table and aligned at the same position are subsequently removed from $\overrightarrow {PE}$. Both the database sequence exhaustion and redundant extension conditions can be detected by checking whether $\overrightarrow {PE} = {\varnothing  }$.

#### Merging paths from the right and left extensions

For a pair $(s^Q ,s^T )$, the paths generated by the right and left extension phases are merged when there exists enough (a pre-set threshold) bridging reads between path pairs. A read is designated as a bridging read if it contains the sequence $s^T$, and with the corresponding prefix being a suffix of the left path and the corresponding suffix being a prefix of the right path. To minimize redundancy in the output set, paths identified by the right and left extensions that are supported by the same set of bridging reads, are first ranked, and subsequently, the corresponding paths in the right and left sets with the same rank are paired and concatenated. For the concatenated paths, new alignment scores are computed by summing the alignment score from both components of the pair (and subtracting the ungapped seed alignment score because it is counted twice), and used to compute the new *E*-values. Left and right paths that cannot be merged are reported individually.

## RESULTS

We benchmarked the performance of GRASP and three other search programs (NCBI's BLASTP and PSI-BLAST, and FASTM) on four datasets (parameters used for the experiments are in Supplementary Data A). For the first two datasets (simulated data), the databases were constructed from known protein family sequences (used to build Pfam profile hidden Markov models), where the ground truth annotation is known. For the third dataset (also simulated data), the database consisted of sequences from a marine metagenome (from 20 organisms), while for the fourth dataset, the database consisted of metagenomic sequences (from a human saliva sample) generated by the human microbiome project (4,38). We generated the simulated data by a random sampling of fragments from the full-length sequences using a Poisson process.

### Datasets

**DS1: Unrelated Pfam families**: The purpose of constructing this dataset was to evaluate whether GRASP could distinguish between reads from different protein families. The database consisted of fragments from three unrelated protein families: PF00154 (recA bacteria recombination protein), PF02834 (LigT like phosphoesterase) and PF04563 (RNA polymerase beta subunit). Short peptide reads (32 amino acids) were sampled from the seed sequences of these Pfam families with expected coverage of 20X; the seed sequences for a Pfam are those family members that were used to generate the multiple sequence alignment from which the Pfam was constructed.
**DS2: Pfam families from the same clan:** To further benchmark GRASP's performance on distinguishing reads from different, but related, protein families, the second dataset was constructed using three Pfam families from the same clan. Such a dataset is challenging because the reference sequences are more similar to each other, and GRASP could have a higher chance to misassemble reads from different families due to their higher interfamily sequence similarity. The database consisted of fragments from members of PF00150 (cellulase), PF01229 (arginine ADP-ribosyltransferase) and PF07745 (glycosyl hydrolase family 53); all of them are from clan CL0058 (Tim barrel glycosyl hydrolase superfamily). Reads were sampled in the same way as for DS1.
**DS3: Simulated marine metagenome:** The third dataset was constructed to evaluate the performance of GRASP on metagenomic datasets, which contain fragments from many protein families, and possibly, contain sequencing errors as well. Genomes of the strains of *Candidatus pelagibacter, Prochlorococcus marinus, Synechococcus, Flavobacteriales, Nitrosococcus oceani, Vibrio, Photobacterium, Erythrobacter, Alteromonas, Roseobacter* and *Shewanella* were used to construct the dataset (relative abundances of these genomes are the same as in a previously described dataset (28)). Nucleotide reads were sampled from these genomes using WGSIM (39) with a read length of 100 bp, an expected coverage of 10X, and an error rate of 1% (for Illumina Technology). The genes (short peptide reads) were then identified using FragGeneScan (16). The resulting database contained 6 273 043 short peptide reads.
**DS4: Real human saliva metagenome**: This dataset was downloaded from the NCBI's Sequence Read Archive (accession number SRS013942). It contained 14 637 415 Illumina reads (after filtering reads of human origin) with a sequence length of 100 bp. FragGeneScan (16) was used to identify short peptide sequences. The resulting database contained 12 036 685 short peptide reads.

### Query protein sequences

Comprehensive sets of query sequences were used in each search experiment, and the results were combined to compute the average (arithmetic mean) performances for each of the four programs. The ‘leave-one-out’ strategy was used for performance evaluation. That is, when a protein sequence is taken as the query, all reads that were sampled from this query protein sequence were removed from the database before conducting the search. For DS1 and DS2, each seed protein sequence for the Pfams was used as a query for the experiments (212 and 161 genes were searched against DS1 and DS2, respectively). For DS3 where there are many more protein families, all genes (16 in total) that are involved in the Glycolysis/Gluconeogenesis pathway (KEGG (40), KO00010) were taken as queries. These genes were chosen from *Dehalococcoides sp. CBDB1*, whose genome was not included in the original genome set used to construct DS3; using this approach we ensure that no reads identical to the query were included in the database, which is consistent with the ‘leave-one-out’ evaluation strategy. In addition, 198 marker genes selected from the Amphora2 (41) dataset were also used in the search experiment to evaluate performance. All marker genes were also taken from *Dehalococcoides sp. CBDB1* for the same reason stated above. Finally, for DS4, genes from the Glycolysis/Gluconeogenesis pathway were used as the queries. The gene set included genes from two organisms with different estimated abundances in human saliva samples, i.e. one from the *Streptococcus* genus (organism code: *SGO*, estimated abundance 14.98% (4)) and the other from the *Lactobacillus* genus (organism code: *LPI*, estimated abundance 0.81% (4)). Glycolysis pathway-related genes from additional genera were also searched against DS4.

### Benchmark measures

Sensitivity (${\rm sen}$) and specificity (${\rm spe}$) for each method was computed as:

\begin{equation*} {\rm sen} = \frac{{{\rm TP}}}{{{\rm TP} + {\rm FN}}}\;{\rm and}\;{\rm spe} = \frac{{{\rm TP}}}{{{\rm TP} + {\rm FP}}} \end{equation*}

where TP, FP and FN are True Positives, False Positives and False Negatives, respectively.

For DS3, where the ground truth annotations are unknown, the reads were annotated in the following way. Each query protein sequence was searched against the full-length genome sequences using TBLASTN. The resulting high-scoring pairs (*E*-value cutoff 10^−3^) were designated as the homologous regions with respect to the query. Then, all reads that were sampled from the genomes, and corresponded to these homologous regions, were deemed as homologous reads. We note that this approach of designating reads as homologs favors BLAST.

There is no ground truth annotation or reference sequence available for the real dataset DS4. Thus, only the specificities and the raw numbers of identified reads (instead of sensitivities) were evaluated for the four programs. To define TP, HMMER3 (42) was used to evaluate whether the identified reads (for BLASTP, PSI-BLAST and FASTM) or the identified contigs (for GRASP) could be assigned to the same protein family as the query. Given a query sequence, its Pfam annotation was extracted from the KEGG database (40), and the Pfam model was aligned with the reads or GRASP contigs using HMMER3 (with default *E*-value cutoff 0.01). For GRASP contigs that could be assigned to the family, all of their constituent reads were taken as TPs. On the other hand when the contigs could not be successfully aligned, their constituent reads were taken as FPs. The specificity measure is defined accordingly.

For each of the three programs (BLASTP, PSI-BLAST and FASTM), a read is considered as being identified by the program as long as it is reported, disregarding the actual length of the alignment between the query and the read. We also assessed the effect of the length of the output alignment on performance (see Supplementary Data C and Supplementary Figure S1). We note that for GRASP contigs, the constituent reads are completely contained in the contigs.

### Performance comparison

**Figure 3.**
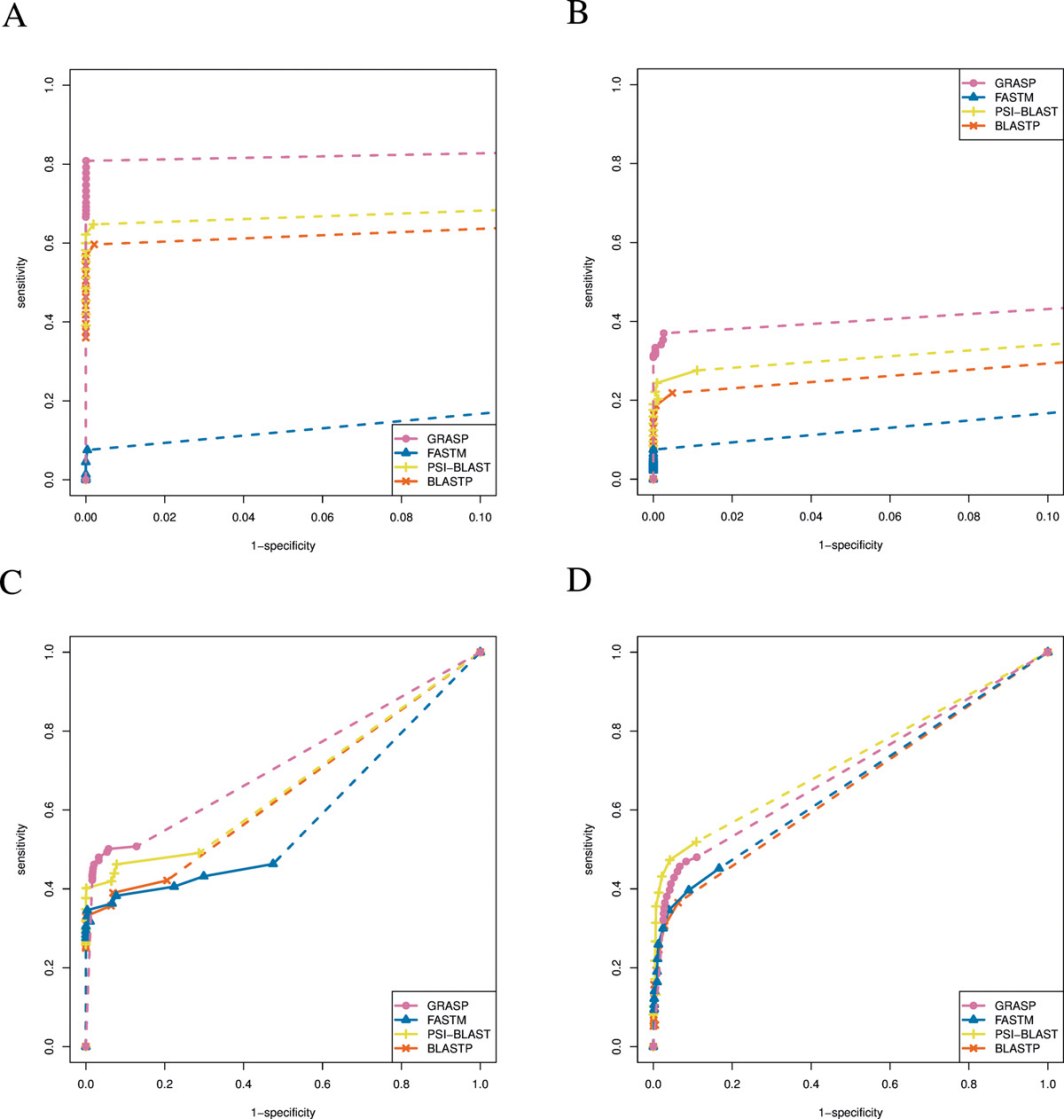
The ROC curves for the performance of GRASP, BLASTP, PSI-BLAST and FASTM on different simulated datasets. (**A**) Pfam-based simulated dataset (DS1) containing three unrelated families. (**B**) Pfam-based simulated dataset (DS2) containing three families from the same clan. (**C**) Simulated marine dataset (DS3) using glycolysis-related genes as queries. (**D**) Simulated marine dataset (DS3) using selected marker genes from Amphora2 as queries. Note that only performances with specificity 90% and higher are shown for (A) and (B). Each individual data point indicates performance at a specific *E*-value cutoff, ranging from 10^−10^ to 10. Dashed lines indicate performances that are extrapolated by projecting to coordinate (1, 1).

The programs were evaluated on all datasets for *E*-value ranges from 10^−10^ to 10. The receiver operating characteristic (ROC) curves for DS1 are shown in Figure 3A. All programs show high specificities even at an *E*-value cutoff of 10 (i.e. GRASP 99.99%, BLASTP 99.78%, PSI-BLAST 99.80% and FASTM 99.96%). All programs also show improved sensitivity with the relaxation of the *E*-value cutoffs (i.e. for the *E*-value cutoff range from 10^−10^ to 10, GRASP's sensitivity increased from 66.60 to 80.83%, for BLASTP from 36.03 to 59.65%, for PSI-BLAST from 39.09 to 64.73% and for FASTM from 0.24 to 7.52%). GRASP shows the best performance among all programs in terms of both specificity and sensitivity. BLASTP and PSI-BLAST show moderate sensitivity and high specificity. FASTM shows relatively lower sensitivity, possibly because it uses regression analysis to infer parameters while computing the *E*-values and thus its performance may be affected by the low complexity and small size of this dataset. The results show that the GRASP algorithm is capable of identifying homologous reads that are otherwise missed (due to the low sequence similarity between them and the query sequence), and can lead to significant sensitivity improvement. Also, the high specificity of GRASP shows that those low sequence identity reads are not placed randomly, but are correctly assembled into contigs using a stringent overlap criterion ($l \ge 10$) for the extension.

The ROC curves for DS2 are shown in Figure 3B. It was observed again that all programs show high specificity even at *E*-value cutoff of 10 (GRASP 99.73%, BLASTP 99.52%, PSI-BLAST 98.89% and FASTM 100%). The specificities for GRASP, BLASTP and PSI-BLAST are lower than that for DS1, potentially because of an increased chance of assembling reads from different families due to high interfamily similarity (recall that all families in DS2 were chosen from the same Pfam clan). FASTM, on the other hand, shows a higher specificity in this experiment. The sensitivity for all programs appears to be lower for this dataset (for the given *E*-value cutoff range, GRASP's sensitivity was increased from 30.91 to 36.98%, for BLASTP from 6.90 to 21.85%, for PSI-BLAST from 8.45 to 27.59% and for FASTM from 2.21 to 7.47%). Nevertheless, GRASP again shows the highest sensitivity among all programs that were tested. The benchmark results on this dataset add evidence that GRASP can assemble and identify homologs with high specificity even for challenging scenarios, and at the same time achieve improved sensitivity.

The ROC curves for searching 16 Glycolysis/Gluconeogenesis-related genes against DS3 are shown in Figure 3C. All four programs show lower specificities compared to those for DS1 and DS2 (for the given *E*-value cutoff range, GRASP's specificity drops from 98.36 to 87.15%, for BLASTP from 100 to 79.40%, for PSI-BLAST from 100 to 71.33% and for FASTM from 99.96 to 52.54%), potentially because of the increased complexity of the read set, sequencing errors and gene finding errors (by the gene-caller). The sensitivities of all programs are lower than that for DS1, but higher than that for the challenging dataset DS2 (for the given *E*-value cutoff range, GRASP's sensitivity was increased from 42.23 to 50.76%, for BLASTP from 25.00 to 42.12%, for PSI-BLAST from 25.92 to 49.09% and for FASTM from 27.56 to 46.30%). GRASP still shows the highest performance in this experiment compared to the other programs. FASTM shows significantly improved performance in this experiment compared to those for DS1 and DS2, potentially because of the more realistic dataset with increased database size and complexity.

The ROC curves for searching Amphora2 marker genes against DS3 are shown in Figure 3D. For specificity within the *E*-value cuotff range (from 10^−10^ to 10), GRASP drops from 97.47 to 88.99%, BLASTP from 99.99 to 93.71%, PSI-BLAST from 99.98 to 89.08% and FASTM from 99.81 to 83.32%; and for sensitivity GRASP increased from 32.14 to 48.02%, BLASTP from 5.22 to 36.50%, PSI-BLAST from 8.13 to 51.92% and FASTM from 9.38 to 45.16%. PSI-BLAST performs the best in this experiment, followed by GRASP. PSI-BLAST's improved performance here is primarily due to the highly conserved nature of the marker genes which when coupled with an iterative detection approach, improves the chances of detecting homologs (using the position-specific matrix generated at the end of the previous round). In fact, if a similar strategy is adopted for GRASP, where reads from the database that are not part of the GRASP output, are mapped to GRASP contigs (as part of an auxiliary read mapping step at the end), then GRASP achieves 67.82% sensitivity and 87.86% specificity at an *E*-value cutoff of 10, and outperforms PSI-BLAST (see Supplementary Data D and Supplementary Figure S2). These results on searching marker genes indicate GRASP's potential applications in microbial community profiling using metagenomic data.

**Figure 4.**
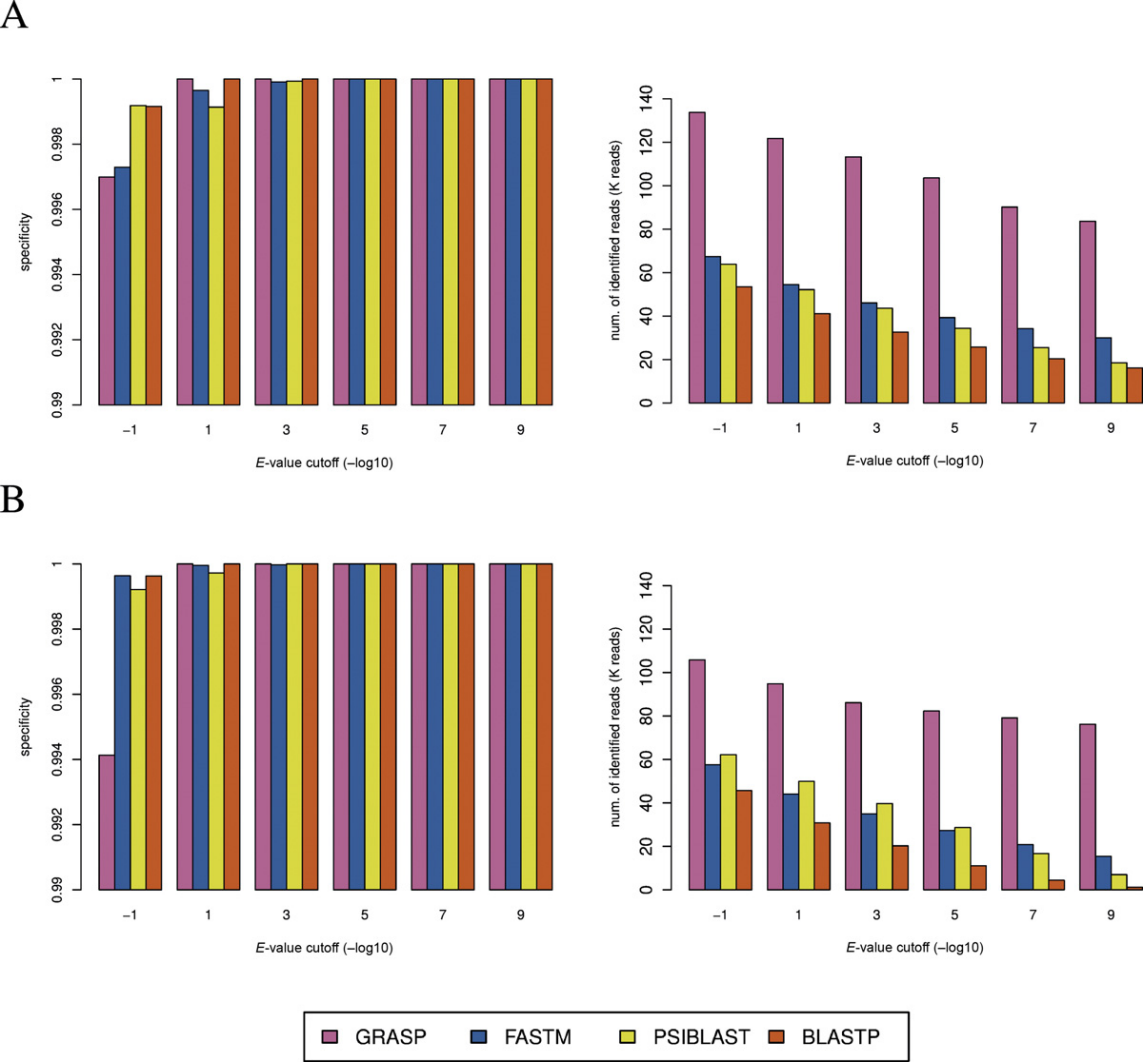
Performance of GRASP, BLASTP, PSI-BLAST and FASTM on a human saliva metagenomic dataset (DS4) using (**A**) *Streptococcus* and (**B**) *Lactobacillus* proteins (Glycolysis/Gluconeogenesis related) as queries, respectively. Left panel: Specificities of the four programs (note that only performances with specificity 99% and higher are shown). Right panel: Number of homologous reads identified by the four programs.

The performance of GRASP and the other search programs on DS4 are shown in Figure 4. The *E*-value cuotffs for this experiment were the same as in the previous ones (10^−10^ to 10), but in the figures we only show the performance on a selected number of cutoffs (due to limited space). The specificity for GRASP is high (>99.40% in the worst case scenario, which is observed for *Lactobacillus* query at *E*-value cutoff of 10) and consistent for both query species, indicating that the majority of assembled contigs can be assigned to the same family as the query. With high specificity, GRASP is able to identify many more homologous reads than the other search programs, which is consistent with the higher sensitivity observation on the simulated datasets. For the given examples, GRASP identified approximately twice more homologous reads than the other three search programs at an *E*-value cutoff of 10. For *E*-value cutoff of 10^−10^, GRASP identified approximately three times more homologous reads than FASTM and at least five times more homologous reads than the BLAST suite. Experiments using genes from other genus (*Prevotella, Fusobacterium* and *Aggregatibacter*) confirm the same trend that GRASP can identify many more homologous reads than the other search programs with high specificity (Supplementary Data E and Supplementary Figure S3). An additional advantage of GRASP is that it makes the most robust prediction at different *E*-value cutoffs, while the other search programs are highly sensitive to the *E*-value cutoff.

### GRASP running time

Since GRASP implements both assembly and search/alignment functions simultaneously, its running time can be expected to be slower than a program that only performs sequence search/alignment. To further investigate its running time, GRASP was run with different query lengths and database sizes (Supplementary Data F). In the first experiment, GRASP was used to search for homologs of queries with different lengths (ranging from ∼200aa to ∼800aa) against a single database (DS4). In the second experiment, GRASP was used to search a single query protein sequence (SGO_0049 with a length of 344aa) against databases with different sizes (constructed by random sampling of reads from DS4). The results of these two experiments are shown in Supplementary Figure S4. It is observed that the actual running time of GRASP grows linearly with respect to the query length when the database is fixed. Furthermore, the running time also scales linearly with the size of the database (for a fixed query). Multithreaded execution mode is also implemented in GRASP, with an empirical speed up of ∼2-fold using four threads observed in these experiments (see Supplementary Figure S4).

## DISCUSSION

In this work we presented GRASP, a query sequence-guided homology detection method that assembles overlapping database reads during the search process. We performed a systematic benchmarking of GRASP and compared it to three other programs that are frequently used for short peptide database searches. In our evaluations using simulated and real datasets, we have shown that GRASP achieves higher sensitivity compared to these three programs, while maintaining a high specificity.

The sensitivity of the basic GRASP approach can be further improved by incorporating an auxiliary read mapping step at the end, which maps the remaining database reads (allowing for mismatches) against contigs output by GRASP. This mapping step improved the sensitivity by ∼10% when searching glycolysis pathway-related genes against DS3, and by ∼20% when searching Amphora2 marker genes against DS3 at a similar specificity level. This observation also motivates a future improvement to the GRASP assembly algorithm that will allow for mismatches in read overlaps during the path extension step; this will also enable the assembly of longer contigs that, in the current implementation, may be fragmented by the premature termination of the extension due to sequence mismatches.

Not surprisingly, the running time of GRASP is more than that of the other three programs, because it also assembles the short peptide fragments during the alignment process. To search for the homologous reads of a protein sequence (SGO_0049) with length 344 (which is close to the average length of a microbial protein) in the saliva sample (∼12 million reads), GRASP takes ∼16 min with a single thread. To improve the scalability of GRASP, we have further implemented GRASP to allow for multithreaded execution; with four threads, the wall clock time reduces to approximately half. In addition, GRASP has flexible parameter settings including the choice of the reduced alphabet and $k$-mer length for assembly seeding, the alignment band size and the termination criteria. These parameters can be tuned to balance the running time and the expected performance (26). For example, by using a reduced alphabet with more equivalent classes and longer seed length, one could expect to improve the running time and specificity but at a decreased sensitivity.

While recent methods have been proposed that have allowed for the *de novo* reconstruction of complete protein sequences directly from short peptide sequences (28), our focus here is only on identifying the homologs of the ‘given’ query/reference protein sequence, and thus is computationally cheaper than a complete assembly of all of the data. GRASP is expected to be important in scenarios where fine-grained analysis is desired. GRASP can be a more accurate substitute for commonly used sequence search programs for the purpose of annotating fragmentary protein sequences in metagenomic datasets, and also allow for improved downstream analysis such as taxonomic profiling and metabolic reconstructions.

## SUPPLEMENTARY DATA

Supplementary Data are available at NAR Online.

SUPPLEMENTARY DATA
