# Supplementary material for: GRASP: Guided Reference-based Assembly of Short Peptides
Source: Nucleic Acids Res. 2014 Nov 20;43(3):e18. doi: 10.1093/nar/gku1210 (PMC4330339; doi:10.1093/nar/gku1210)
Supplement: SUPPLEMENTARY DATA [file supp_43_3_e18__index.html]

GRASP: Guided Reference-based Assembly of Short Peptides — GRASP: Guided Reference-based Assembly of Short Peptides — SUPPLEMENTARY DATA 

# GRASP: Guided Reference-based Assembly of Short Peptides

## SUPPLEMENTARY DATA

**Files in this Data Supplement:**

- SUPPLEMENTARY DATA
