# Supplementary material for: GRASP: Guided Reference-based Assembly of Short Peptides
Source: Nucleic Acids Res. 2014 Nov 20;43(3):e18. doi: 10.1093/nar/gku1210 (PMC4330339; doi:10.1093/nar/gku1210)
Supplement: SUPPLEMENTARY DATA [file supp_gku1210_nar-02278-met-z-2014-File006.pdf]

Supplementary Data for
"GRASP: Guided Reference-based Assembly of Shorted Peptides"

Cuncong Zhong, Youngik Yang, and Shibu Yooseph*
Informatics Department, J. Craig Venter Institute, La Jolla, CA 92037, USA.
*Corresponding author

A. Parameters

All experimental results were generated using the default set of parameters [for NCBI BLAST (BLASTP and PSI-BLAST) suite (1,2), FASTM (3), and GRASP], or otherwise detailed as follows. The performances of all programs were tested under different E-value cutoffs, ranging from 10^{-10} (high specificity) to 10 (high sensitivity), to generate the ROC curves shown in the main text and in this supplement. PSI-BLAST was run with 3 iterations. FASTM was run using the BLOSUM62 scoring matrix with gap open and extension penalties of -11 and -1, respectively (using the '-s BL62' option), to match the settings used by the other programs.

The GRASP default parameters were set as the follows. For seeding, the GBMR10 alphabet and a seed length of 6 amino acids were used. These seeding parameters were chosen based on their overall performance in terms of selectivity and sensitivity [see Figure 2 in reference (4)]. The actual alignment score of the seed pair was computed under the original amino acid alphabet. And such an alignment score was further used to filter low-similarity seed pairs (default alignment score cutoff $0.6*a$, where a is the average of the matching scores for the same type of amino acids, i.e. scores in the diagonals, in the given scoring matrix). For the assembly module, 10 amino acids ($l = 10$) were required as the minimum overlap to define the supporting reads r of a given path p. Also, at least 3 supporting reads were required to extend a path. The score drop-off threshold used to terminate the extension is 25 (bit-score). For the alignment module, the BLOSUM62 matrix and a gap open and gap extension penalty of -11 and -1, respectively, were used for all experiments. The same set of Karlin-Altschul statistics (5) parameters in the BLAST suite was used in GRASP to compute E-values. The band size for the alignment is 40.

B. GRASP pseudo-code

The GRASP algorithm contains two major components. The first component (**GRASP_MAIN**) identifies the initial seeds and issues the extension calls in both left and right directions. The second component (**EXTEND**) extends the path with the best existing maximal extension sequence in the priority queue, and reinserts the newly identified maximal extension sequences, along with their corresponding assemblies, back to the priority queue based on their coverages (the number of supporting reads). The pseudo-codes for both components are detailed in this supplement. For notations please refer to the main text.

GRASP_MAIN(Q , R)

$\sum^*$ $\leftarrow$ user specified reduced alphabet
build suffix array SA on R ; build suffix array rSA on $rev(R)$
build hash table H using all n where n is a k -long substring of some $r \in R$
// the key of H is n^{Σ^*} (a k -mer in alphabet $\sum^*$), and the value is the set N of k -mers in alphabet $\sum$, where for
// each $n \in N$ we have $reduced(n) = n^{\Sigma^*}$
for each m where m is a k -long substring of Q **do:**
  $q \leftarrow m$
  for each $n \in H[reduced(m)]$ **do:**   // $reduced(n) = reduced(m)$ indicates a seed match in $\sum^*$
    $p \leftarrow n$
    // extension to the C-terminus (right extension)
    align q and p // tradition Needleman-Wunsch algorithm
    T^{DP} $\leftarrow$ dynamic programming table of the alignment between q and p
    A^R $\leftarrow$ alignment structure constructed on (right, q , p , T^{DP} , SA , rSA)
    PQ^R $\leftarrow \varnothing$ // a priority queue that holds all possible extensions
    ENQUEUE(PQ^R, A^R)   // insert A^R into priority queue PQ^R
    while $PQ^R != \varnothing$ **do**:
      call EXTEND(PQ^R)
    endwhile
    // extension to the N-terminus (left extension)
    align $rev(q)$ and $rev(p)$ // traditional Needleman-Wunsch algorithm
    rT^{DP} $\leftarrow$ dynamic programming table of the alignment between $rev(q)$ and $rev(p)$
    A^L $\leftarrow$ alignment structure constructed on (left, $rev(q)$, $rev(p)$, rT^{DP} , rSA , SA)
    PQ^L $\leftarrow \varnothing$ // a priority queue that holds all possible extensions
    ENQUEUE(PQ^L, A^L)
    while $PQ^L != \varnothing$ **do**:
      call EXTEND(PQ^L)
    endwhile
    connect right and left extension paths if enough bridging reads are present
    output assembled paths and corresponding reads
  endfor
endfor
return

EXTEND(PQ)

$A \leftarrow$ DEQUEUE(PQ) // take the alignment that has the highest priority in the queue

// unpack the alignment information

$direction \leftarrow A.direction$; $p \leftarrow A.p$; $q \leftarrow A.q$

$T^{DP} \leftarrow A.T^{DP}$; $SA \leftarrow A.SA$; $rSA \leftarrow A.rSA$

$x \leftarrow$ l -long suffix of p // if $|p| < l$ then take $x = p$

// search the reversed suffix array to determine valid supporting reads

$[g', h'] \leftarrow$ suffix array range returned by searching $rev(x)$ against rSA

$mh' \leftarrow h'$ // records the last successfully returned position

$lb \leftarrow 1$; $rb \leftarrow |p| - l$ // left and right bound for the binary search on the prefix length

while $lb < rb$ **do:**

$e \leftarrow (\frac{rb - lb + 1}{2} + l)$ -long suffix of p

$[a, b] \leftarrow$ search $rev(e)$ against $rSA[g', mh']$

if $a \le b$ **do:** // a valid range is returned means the suffix is in the database, search proceeds with longer suffix

if $mh' < b$ **do:**

$mh' \leftarrow b$

endif

$rb = rb - (\frac{rb - lb + 1}{2})$

else: // an invalid range means the suffix is not in the database, search proceeds with shorter suffix

$lb = lb + (\frac{rb - lb + 1}{2})$

endif

endwhile

$U \leftarrow$ a set that contains all read IDs whose suffixes are recorded within the range $rSA[g', mh']$

// select maximal extension sequence and extend the assembly

$[g, h] \leftarrow$ suffix array range returned by searching x against SA

while $g \le h$ **and** $SA[g].rid \in U$ **do:** // $SA[g].rid$ is the read ID

$g{+}{+}$ // jump to the first valid supporting read

endwhile

// initialization: $begin$ means the starting position of the suffix array partition, $last\_valid$ means the end of it

// $invalid\_array[i]$ records number of invalid suffixes between position g and i , inclusively

$begin \leftarrow g$; $last\_valid \leftarrow g$; $invalid\_array[g] \leftarrow 0$

$i \leftarrow g + 1$

(Continue on the next page)

EXTEND(PQ) continue

(Continue from the previous page)

while $i \le h+1$ **do:**
  if $LCP[i] < |SA[i-1]|$ **do:** // compute the number of supporting reads in the partition
    $num\_suffixes \leftarrow last\_valid - begin + 1$
    $num\_invalid \leftarrow invalid\_array[last\_valid] - invalid\_array[prev]$
    $num\_sr \leftarrow num\_suffixes - num\_invalid$
    if $num\_sr \ge c$ **do:** // c is the cutoff for minimum number of supporting reads
      $p' \leftarrow p \bullet$ suffix of $SA[i-1]$ that follows its prefix x
      $q' \leftarrow$ substring of Q that is extended from q to reach a length of $|p'| + \frac{d}{2}$ // d is the band size
      reinitialize alignment between q' and p' using T^{DP}
      $T^{DP}{}' \leftarrow$ dynamic programming table of the alignment between q' and p'
      if score drop-off is above the pre-set threshold **do:**
        return
      else:
        $A^{next} \leftarrow$ alignment structure constructed on ($direction$, q' , p' , $T^{DP}{}'$, SA , rSA)
        ENQUEUE(PQ, A^{next}) // priority computed based on num_sr
      endif
    endif
    while $i \le y+1$ **and** $SA[i].rid \notin U$ **do:** // jumps to the next valid read
      $invalid\_array[i] \leftarrow invalid\_array[i-1]+1$; $i++$
    endwhile
    // reset the indexes for a new partition
    $begin \leftarrow i$; $last\_valid \leftarrow i$; $invalid\_array[i] \leftarrow invalid\_array[i-1]$
  else: // in case of expansion of the current partition
    if $SA[i].rid \notin U$ **do:**
      $invalid\_array[i] \leftarrow invalid\_array[i-1]+1$
    else: // expand the current partition by increasing the end index of the partition, i.e. $last\_valid$
      $last\_valid \leftarrow i$; $invalid\_array[i] \leftarrow invalid\_array[i-1]$
    endif
  endif
  $i++$
endwhile
return

C. Evaluation of impact of different length cutoffs

BLASTP results were interpreted as three different sets to evaluate the impact of using different length constraints for filtering the local alignment-based search results. The set "BLASTP (full)" was used to represents those reads whose full-length sequences were aligned by BLASTP, "BLASTP (partial 50%)" for those who had >50% of their full-length sequences aligned by BLASTP, and "BLASTP (partial)" for all reads that had any part of their sequences aligned by BLASTP. The performances of BLASTP for these three interpretations are shown in Supplementary Figure S1. It is observed that "BLASTP (full)" shows slightly improved specificity but significantly lower sensitivity compared to the other two, and "BLASTP (partial)" and "BLASTP (partial 50%)" show no significant difference from each other in terms of both specificity and sensitivity.

D. Auxiliary read mapping step at the end of GRASP

The path extension module of GRASP does not consider mismatches or gaps in the sequence. Even though it is possible to take mismatches into account during extension by issuing multiple suffix array searches (4), the current GRASP setting is conceptually straightforward and computationally efficient, and is capable of reducing the redundancy of the output contigs. GRASP only outputs those reads that perfectly match some substring of the assembled contigs. Here we show that the performance of GRASP can be further improved by incorporating an auxiliary read mapping step at the end, using the simulated marine data set (DS3) as an example.

The auxiliary read mapping step aims at recruiting the reads that are not detected by the main GRASP algorithm due to mismatches. The read mapping step works as follows. For each query protein sequence, we mapped remaining reads in the database against the output contigs. We mapped a read if >60% of its full-length sequence can be aligned to one of the contigs with at most 3 mismatches (only substitution is considered). Note that we allowed up to 3 mismatches because DS3 was generated with 1% error rate, which is higher than the empirical error rate for the Illumina technology (>85% of the reads have error rate less than 0.1%, see http://res.illumina.com/documents/products/technotes/technote_q-scores.pdf). Supplementary Figure S2A shows the performances of GRASP+mapping and the other three programs in searching 16 glycolysis pathway-related genes in *Dehalococcoides sp. CBDB1* against DS3. As compared to the performances shown in Figure 3C of the main text, incorporating the read mapping step further improves GRASP's sensitivity by >10% with a slight decrease in specificity (GRASP+mapping achieves 62.62% sensitivity and 86.57% specificity with an E-value cutoff of 10, and GRASP without mapping achieves 50.76% sensitivity and 87.15% specificity with the same E-value cutoff). Supplementary Figure S2B shows the performances of GRASP+mapping and the other three programs in searching the 198 Amphroa2 (6) marker genes in *Dehalococcoides sp. CBDB1*. As compared to the results shown in Figure 3D in the main text, GRASP+mapping achieves ~20% higher sensitivity but ~1% lower specificity (GRASP+mapping achieves 67.82% sensitivity and 87.86% specificity with an E-value cutoff of 10, and GRASP without mapping achieves 48.02% sensitivity and 88.99% specificity with the same E-value cutoff). Both results show that incorporating the auxiliary read mapping step will significantly improve GRASP's sensitivity at the same specificity level. The running time for the auxiliary read mapping step is minimal compared to the main program, as we can assume near-perfect matches between the contigs and the reads.

E. Performance benchmark on searching DS4 with other queries

Supplementary Figure S3 shows the performances of the four programs (GRASP, FASTM, PSI-BLAST, and BLASTP) in searching *Prevotella* (organism code: *PIT*, estimated abundance 12.03%), *Fusobacterium* (organism code: *FUS*, estimated abundance 6.39%), and *Aggregatibacter* (organism code: *AAP*, estimated abundance 1.42%) against the real HMP (Human Microbiome Project) saliva data set (i.e. DS4). For definitions of specificity and true homologous reads please refer to the main text. The results shown in Supplementary Figure S3 further confirm the observation that GRASP is capable of recruiting more true homologous reads than the other search programs with a high specificity.

F. GRASP run-time

GRASP runs slower than the NCBI BLAST suite and FASTM in most of the experiments. This is expected since GRASP also performs *de novo* assembly along with the alignment. Supplementary Figure S4 shows the run-time for GRASP on different queries and targets (databases). The run-time variation in Supplementary Figure S4A (i.e. different queries against same database) is more significant than that in Supplementary Figure S4B (same query against different databases), because the actual amount of assemblies/alignments to be performed is query-specific (e.g. the number of seeds that can be identified from the query to initiate the assemblies/alignments). Both results show linear correlations between the run-time and query/database size. It implies a linear run-time growth with respect to the number of proteins being searched (in most real applications, the database size is fixed for a given metagenomic dataset). Running GRASP in multi-threaded mode is capable of improving the actual run-time (i.e. 2.12 fold speedup was observed with 4 threads, see Supplementary Figure S4). Potential overhead was observed due to the inter-thread communications that are required to avoid redundant extension of reads that have been consumed by other extensions.

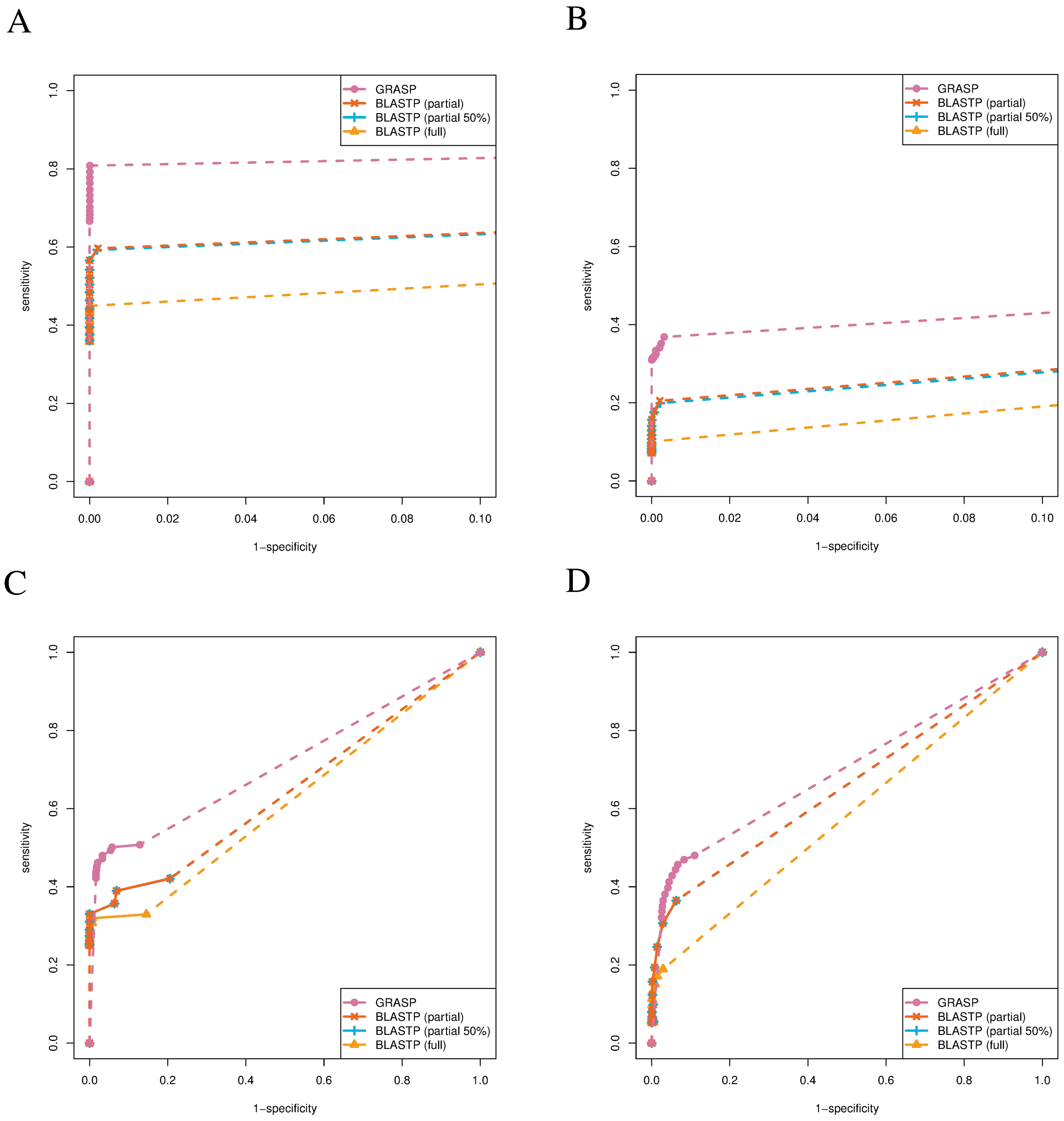

Supplementary Figure S1: The ROC curves for the performances of GRASP and BLASTP (with different length constraints) on different simulated datasets. (A) Pfam simulated data set containing three unrelated families, i.e. DS1. (B) Pfam simulated data set containing three families from the same clan, i.e. DS2. (C) Simulated marine data set, i.e. DS3, using glycolysis related genes as queries. (D) Simulated marine data set, i.e. DS3, using selected marker genes from Amphora2 as queries. Note that only performances with specificity 90% and higher are shown for (A) and (B). Each individual data point indicates performance at a specific E-value cutoff, ranging from 10^{-10} to 10. Dashed lines indicate performances that are extrapolated by projecting to coordinates (0, 0) and (1, 1).

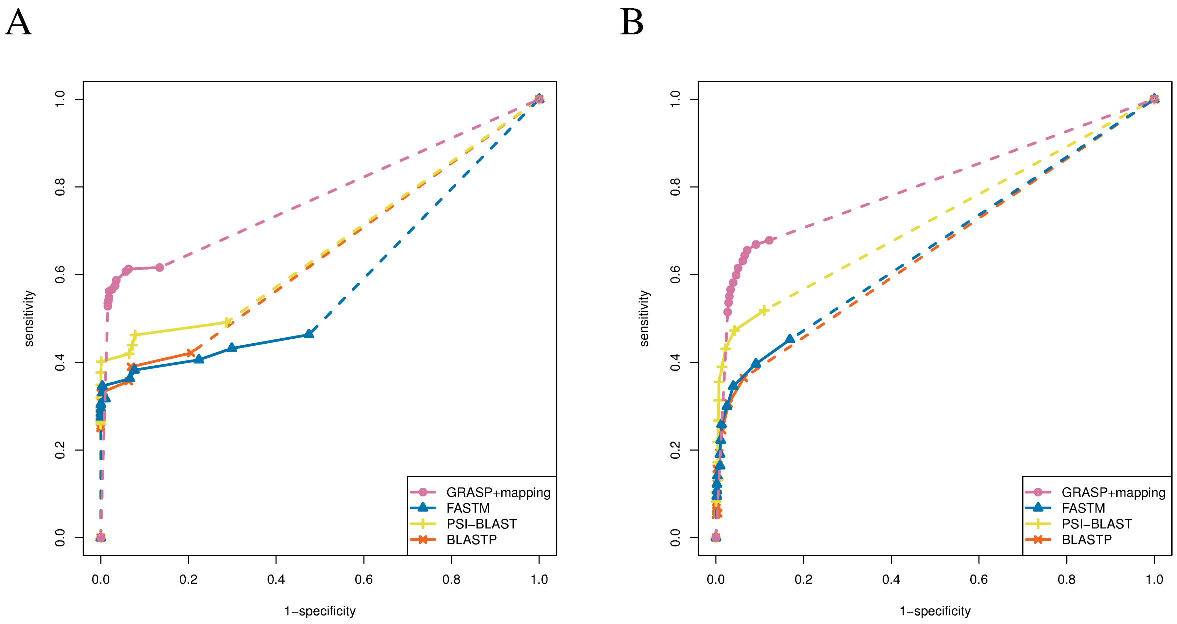

Supplementary Figure S2: Performances of GRASP+mapping, FASTM, PSI-BLAST, and BLASTP in searching the simulated marine data set (DS3). (A) Performances with 16 glycolysis pathway-related genes as queries. (B) Performances with 198 Amphroa2 marker genes as queries. All genes were selected from *Dehalococcoides sp. CBDB1*.

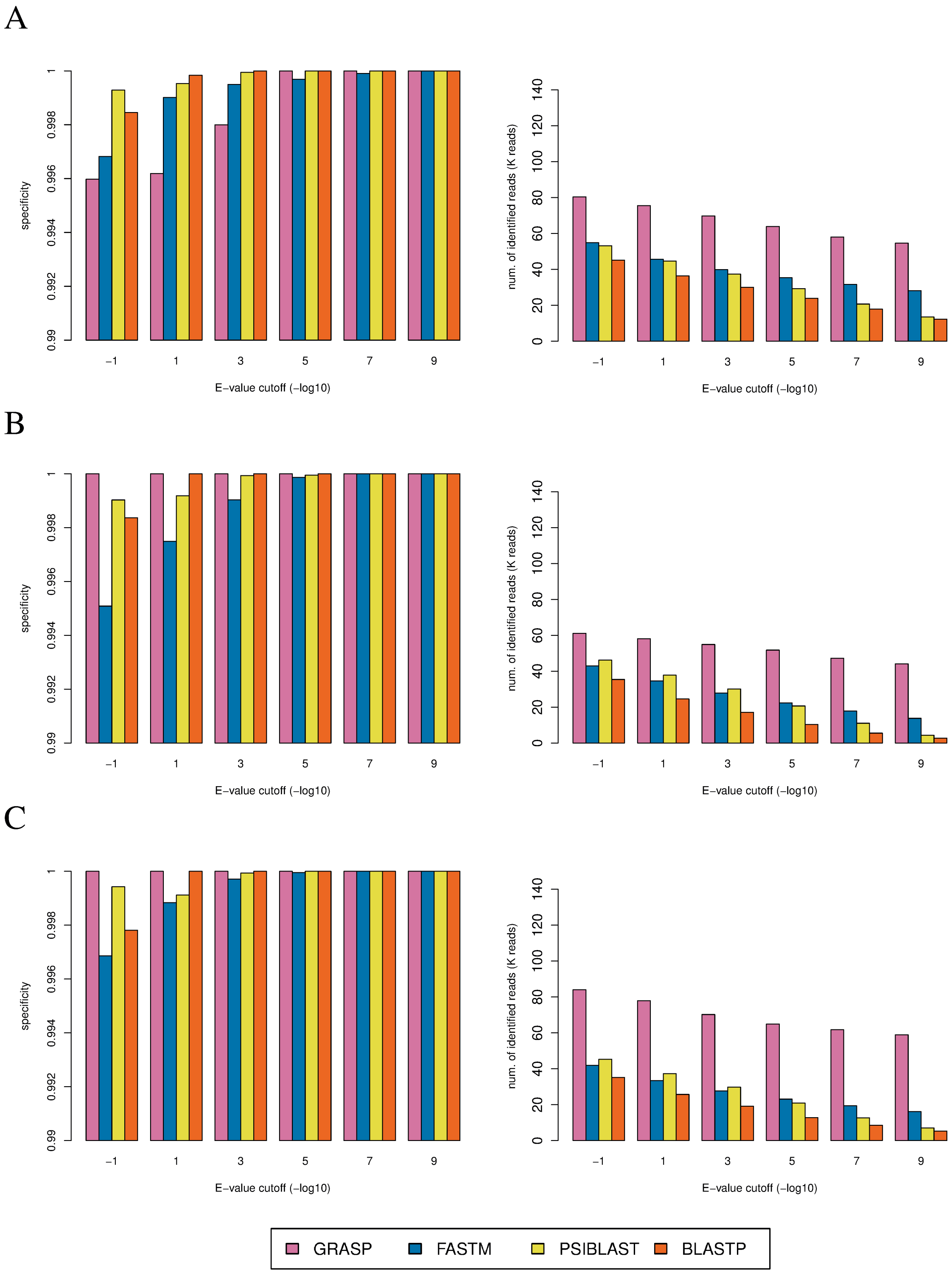

Supplementary Figure S3: Performances of GRASP, FASTM, PSI-BLAST, and BLASTP in searching the glycolysis related genes in (A) *Prevotella*, (B) *Fusobacterium*, and (C) *Aggregatibacter* against the real HMP saliva metagenomic data set (i.e. DS4). The left panel shows the specificities and the right panel shows the raw number of recruited true homologous reads. All programs show high specificities in the experiment (>99%). GRASP recruits the highest number of true homologous reads.

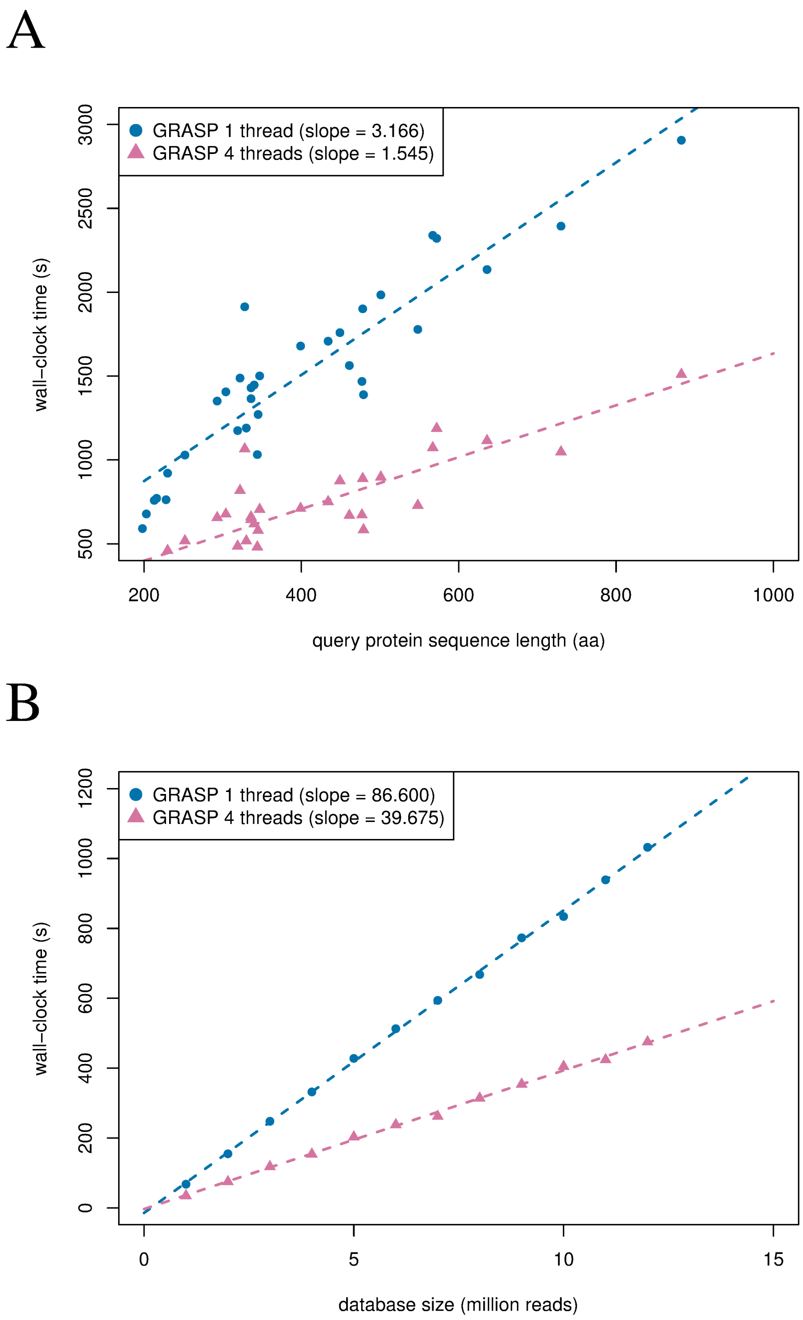

Supplementary Figure S4: The run-time of GRASP. (A) GRASP run-time for searching 33 query genes (glycolysis-related genes in *Streptococcus SGO*) with different lengths against DS4 (12,036,685 short peptide reads, translated from Illumina nucleotide reads with length of 100 bp). (B) GRASP run-time for searching a single gene (SGO_0049, length 344 aa) against databases with different sizes (databases constructed by random sampling reads from DS4). The average speedup ratio of using 4-threads, computed based on linear regression, is 2.12 fold.

References

1. Altschul, S.F., Gish, W., Miller, W., Myers, E.W. and Lipman, D.J. (1990) Basic local alignment search tool. *Journal of molecular biology*, **215**, 403-410.
2. Altschul, S.F., Madden, T.L., Schaffer, A.A., Zhang, J., Zhang, Z., Miller, W. and Lipman, D.J. (1997) Gapped BLAST and PSI-BLAST: a new generation of protein database search programs. *Nucleic acids research*, **25**, 3389-3402.
3. Mackey, A.J., Haystead, T.A. and Pearson, W.R. (2002) Getting more from less: algorithms for rapid protein identification with multiple short peptide sequences. *Molecular & cellular proteomics : MCP*, **1**, 139-147.
4. Ye, Y., Choi, J.H. and Tang, H. (2011) RAPSearch: a fast protein similarity search tool for short reads. *BMC bioinformatics*, **12**, 159.
5. Karlin, S. and Altschul, S.F. (1990) Methods for assessing the statistical significance of molecular sequence features by using general scoring schemes. *Proceedings of the National Academy of Sciences of the United States of America*, **87**, 2264-2268.
6. Wu, M. and Scott, A.J. (2012) Phylogenomic analysis of bacterial and archaeal sequences with AMPHORA2. *Bioinformatics*, **28**, 1033-1034.
